# Simulated microgravity promotes the formation of tridimensional cultures and stimulates pluripotency and a glycolytic metabolism in human hepatic and biliary tree stem/progenitor cells

**DOI:** 10.1038/s41598-019-41908-5

**Published:** 2019-04-03

**Authors:** Daniele Costantini, Diletta Overi, Luca Casadei, Vincenzo Cardinale, Lorenzo Nevi, Guido Carpino, Sabina Di Matteo, Samira Safarikia, Mariacristina Valerio, Fabio Melandro, Mariano Bizzarri, Cesare Manetti, Pasquale Bartolomeo Berloco, Eugenio Gaudio, Domenico Alvaro

**Affiliations:** 1 Department of Medicine and Medical Specialties, Sapienza University of Rome, Rome, Italy; 2 Division of Human Anatomy, Department of Anatomical, Histological, Forensic Medicine and Orthopedics Sciences, Sapienza University of Rome, Rome, Italy; 3 Department of Chemistry, Sapienza University of Rome, Rome, Italy; 4 Department of Medico-Surgical Sciences and Biotechnologies, Polo Pontino, Sapienza University of Rome, Rome, Italy; 5 Department of Movement, Human and Health Sciences, Division of Health Sciences, University of Rome “Foro Italico”, Rome, Italy; 6 Department of General Surgery and Organ Transplantation, Sapienza University of Rome, Rome, Italy; 7 Department of Experimental Medicine, Sapienza University of Rome, Rome, Italy

## Abstract

Many pivotal biological cell processes are affected by gravity. The aim of our study was to evaluate biological and functional effects, differentiation potential and exo-metabolome profile of simulated microgravity (SMG) on human hepatic cell line (HepG2) and human biliary tree stem/progenitor cells (hBTSCs). Both hBTSCs and HepG2 were cultured in a weightless and protected environment SGM produced by the Rotary Cell Culture System (Synthecon) and control condition in normal gravity (NG). Self-replication and differentiation toward mature cells were determined by culturing hBTSCs in Kubota’s Medium (KM) and in hormonally defined medium (HDM) tailored for hepatocyte differentiation. The effects on the expression and cell exo-metabolome profiles of SMG versus NG cultures were analyzed. SMG promotes tridimensional (3D) cultures of hBTSCs and HepG2. Significative increase of stemness gene expression (p < 0.05) has been observed in hBTSCs cultured in SMG when compared to NG condition. At the same time, the expression of hepatocyte lineage markers in hBTSCs differentiated by HDM was significantly lower (p < 0.05) in SMG compared to NG, demonstrating an impaired capability of hBTSCs to differentiate *in vitro* toward mature hepatocytes when cultured in SMG condition. Furthermore, in HepG2 cells the SMG caused a lower (p < 0.05 vs controls) transcription of CYP3A4, a marker of late-stage (i.e. Zone 3) hepatocytes. Exo-metabolome NMR-analysis showed that both cell cultures consumed a higher amount of glucose and lower glutamate in SMG respect to NG (p < 0.05). Moreover, hBTSCs media cultures resulted richer of released fermentation (lactate, acetate) and ketogenesis products (B-hydroxybutyrate) in SGM (p < 0.05) than NG. While, HepG2 cells showed higher consumption of amino acids and release of ketoacids (3-Methyl-2-oxovalerate, 2-oxo-4-methyl-valerate) and formiate with respect to normogravity condition (p < 0.05). Based on our results, SMG could be helpful for developing hBTSCs-derived liver devices. In conclusion, SMG favored the formation of hBTSCs and HepG2 3D cultures and the maintenance of stemness contrasting cell differentiation; these effects being associated with stimulation of glycolytic metabolism. Interestingly, the impact of SMG on stem cell biology should be taken into consideration for workers involved in space medicine programs.

## Introduction

Gravity exerts a remarkable impact on cell biology, especially on metabolism and cytoskeleton^1^. Indeed, SMG has been proved to profoundly influence both function and phenotypic determination of mature and stem cells^2^ since in absence of proper physical constraints, cells are unable to find a unique, specific differentiated fate^3^. Exposure of embryonic stem cells (ES) to SMG for 15 days blocked cell differentiation with ES ‘frozen’ into a pluripotent phenotype and their gene-expression pattern (Sox1 and Sox2) was similar to control ES at the very early stages of development^4^. Furthermore, human bone marrow stem cells (BMSC) have been shown to largely remain in an undifferentiated state after exposure to SMG for three days^5^ or are reverted to a pluripotent state when committed towards differentiation^6^. Moreover, SMG hinders the differentiating pathways in BMSC with reduction of osteogenic marker expression and incomplete maturation of the bone microenvironment^7^, eventually driving cells towards unexpected cell fate specification (i.e., adipocyte fate)^8,9^. SMG delayed also hematopoietic stem cells (HSC) differentiation into committed progenitor cells when HSC are exposed to weightlessness for longer periods (11 days)^9^. Even cancer stem cells can be ‘arrested’ in their phenotypic differentiating pathways^10^ by SMG that also may induce epithelial-to-mesenchymal transition, as observed in human keratinocytes^11^. In substance a robust scientific background suggests that gravity represents a key regulator of the cell destiny.

Liver is the main metabolic organ of the organism and it is conceivably vulnerable to space-derived lesions or dysfunctions. A population of stem cells, the *human biliary tree stem/progenitor cells* (hBTSCs), was identified at the bottom of peribiliary glands (PBG), with a great concentration into the common hepatic duct, cystic duct, hepatopancreatic ampulla. hBTSCs progressively lose their molecular characteristics and phenotype at the lumen surface, assuming mature features^12^. These cells express endodermal stem markers (OCT4, Nanog, EpCAM, LGR5, SOX9, SOX17, PDX1) and they can differentiate *in vivo* and *in vitro* in mature hepatocytes, cholangiocytes and pancreatic islets^13,14^. hBTSCs were demonstrated to be a valid vehicle for regenerative medicine because their features and availability^15^. In this study we evaluated the effects of SMG on biological properties and functions of cultured human hepatic and biliary tree stem/progenitor cells, on their differentiation potential and exo-metabolome profile, to evaluate whether SMG may help the development of tridimensional cultures of human biliary tree stem cells (hBTSCs), to be used for the regenerative medicine of liver diseases and for development of liver devices.

## Results

### Tridimensional cultures of hBTSCs and HepG2 cells

**Figure 1:**
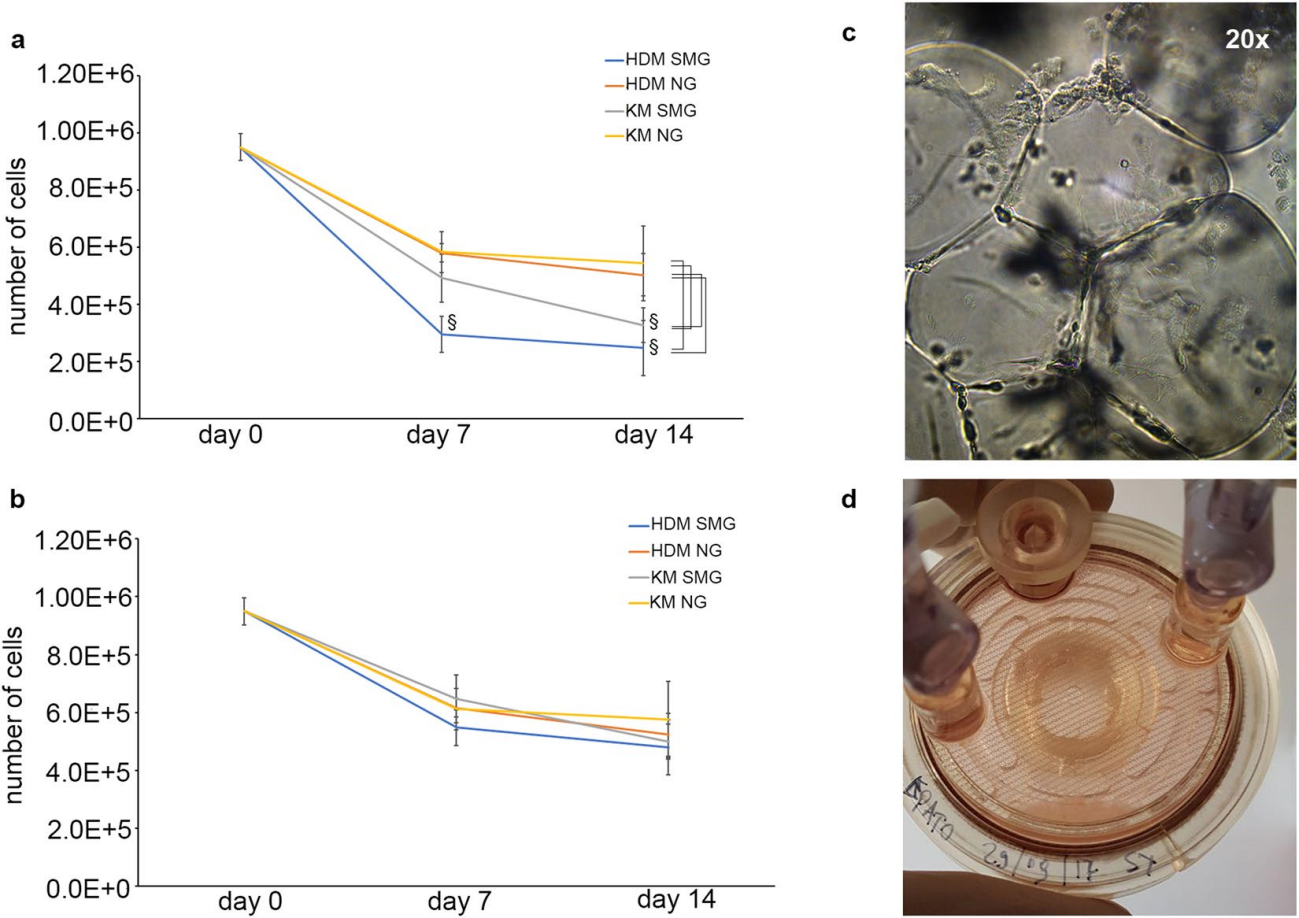
Viability of tridimensional hBTSCs cultures in Kubota’s Medium (KM) and in hormonally defined medium (HDM) both in simulated microgravity (SMG) and in normal gravity condition (NG). hBTSCs viability was analyzed at day 0, at 7 days and at 14 days. The number of alive cells significantly decreased in SMG at 7 and 14 days when they were cultured in absence of an adhesion substrate (^§^p < 0.01; n = 3) (**a**). No significant differences in cells number were observed when hBTSCs were cultured in presence of Cytodex 3 microcarriers in SMG compared to controls in NG. Cytodex 3 microcarriers provided a proper and inert surface to grant adhesions signals that are necessary to ensure epithelial cell growth (**b**). Bright field image of hBTSCs grown on Cytodex 3 in SMG at 14 days (10x) (**c**). hBTSCs in presence of Cytodex 3 formed naked-eyes visible tridimensional cultures in a vessel at 14 days of culture (**d**).

When cultured in SMG, hBTSCs or HepG2 grew as spheroids and showed a decreased number of viable cells in the presence of basal and differentiation media at 7–14 days in comparison with control cells in NG (*p* < 0.01 at 14 days) (Fig. 1a). On the contrary, no significant differences with respect to NG were observed in the number of viable cells when they were cultured adherent to the surface of Cytodex 3 microcarriers in SMG (Fig. 1b). The use of Cytodex 3 microcarrier beads (N = 4) minimizes cell loss and increases cell viability in SMG condition. Indeed, for both hBTSCs and HepG2 cells, cultured in presence of Cytodex 3 microcarriers, SMG condition promoted the formation of tridimensional clusters, as showed in Fig. 1c and Supplementary videos 1 and 2, which could be also visible to the naked eye (Fig. 1d). In addition, cells were able to recreate stable two-dimension cultures once they were re-plated in NG.

### Analysis of the expression in hBTSCs and HepG2 cells cultured in normogravity and SMG

**Figure 2:**
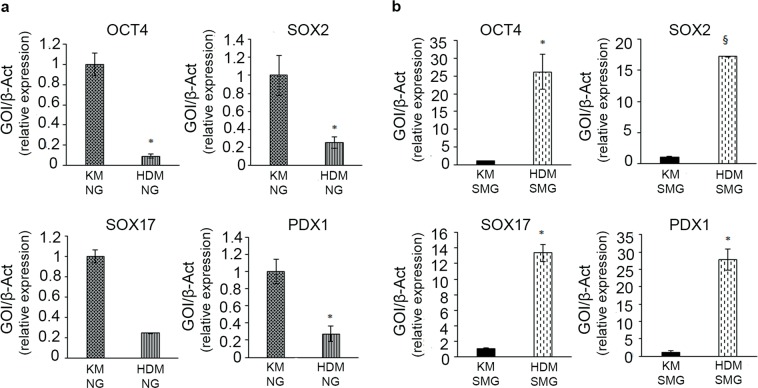
Up-regulation of typical stem cell markers in simulated microgravity (SMG). Levels of gene expression for the stem cell phenotype (OCT4, SOX2, SOX17, PDX1) in hBTSCs cultured in presence of Kubota’s Medium (KM) and hormonally defined medium (HDM) on Cytodex 3 microcarriers in normal gravity condition (NG, control) (**a**) and in SMG (*p < 0.05; ^§^p < 0.01; n = 4) (**b**).

**Figure 3:**
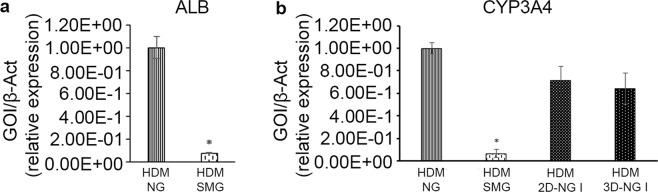
Transcriptional analysis of mature hepatocyte markers in hBTSCs in simulated microgravity (SMG). The gene expression of ALB in hBTSCs cultured in SMG on Cytodex 3 microcarriers in presence of HDM compared to the expression of the same cells in NG (*p < 0.05; n = 4) (**a**). Levels of CYP3A4 significantly dropped down when hBTSCs were cultured in HDM in SMG (*p < 0.05; n = 4). They were restored when cells were put back in NG conditions, both cultured as a monolayer (2D) and on Cytodex 3 microcarriers (3D) (**b**).

The expression of stem cell genes (OCT4, SOX2, SOX17, PDX1) was tested in hBTSCs and HepG2 cells cultured with Cytodex 3 microbeads in SMG and in NG condition and in either Kubota’s Medium (KM) or hormonally defined medium (HDM). In NG condition, the expression of the stem cell genes OCT4, SOX2, SOX17, and PDX1 resulted markedly reduced (25 folds for OCT4; 9 folds for SOX2; 7.7 folds for SOX17; 6.7 folds for PDX1) after 15 days in HDM in comparison with hBTSCs maintained in KM (*p* < 0.05) (Fig. 2a). In contrast, in cells cultured for 15 days in SMG, the expression of stem cell genes was preserved or even increased (an increase of 26.17 folds for OCT4; 13.37 folds for SOX2; 27.73 folds for SOX17; 17.24 folds for PDX1) after culture in HDM than in KM (*p* < 0.05) (Fig. 2b). In sum, SMG maintained hBTSCs in an undifferentiated state even when they were incubated in HDM medium that normally induced differentiation toward mature hepatocytes in NG. In keeping, the expression of typical mature hepatocyte genes (ALB, CYP3A4) in hBTSCs grown in HDM was markedly higher in NG than in SMG (*p* < 0.05) (Fig. 3a,b). After the culturing in SMG, the same cells were replaced for 15 days in NG condition (see Supplementary Fig. 2), which resulted in the restoration of high level of CYP3A4 gene expression in the differentiation condition (HDM) (Fig. 3b).

**Figure 4:**
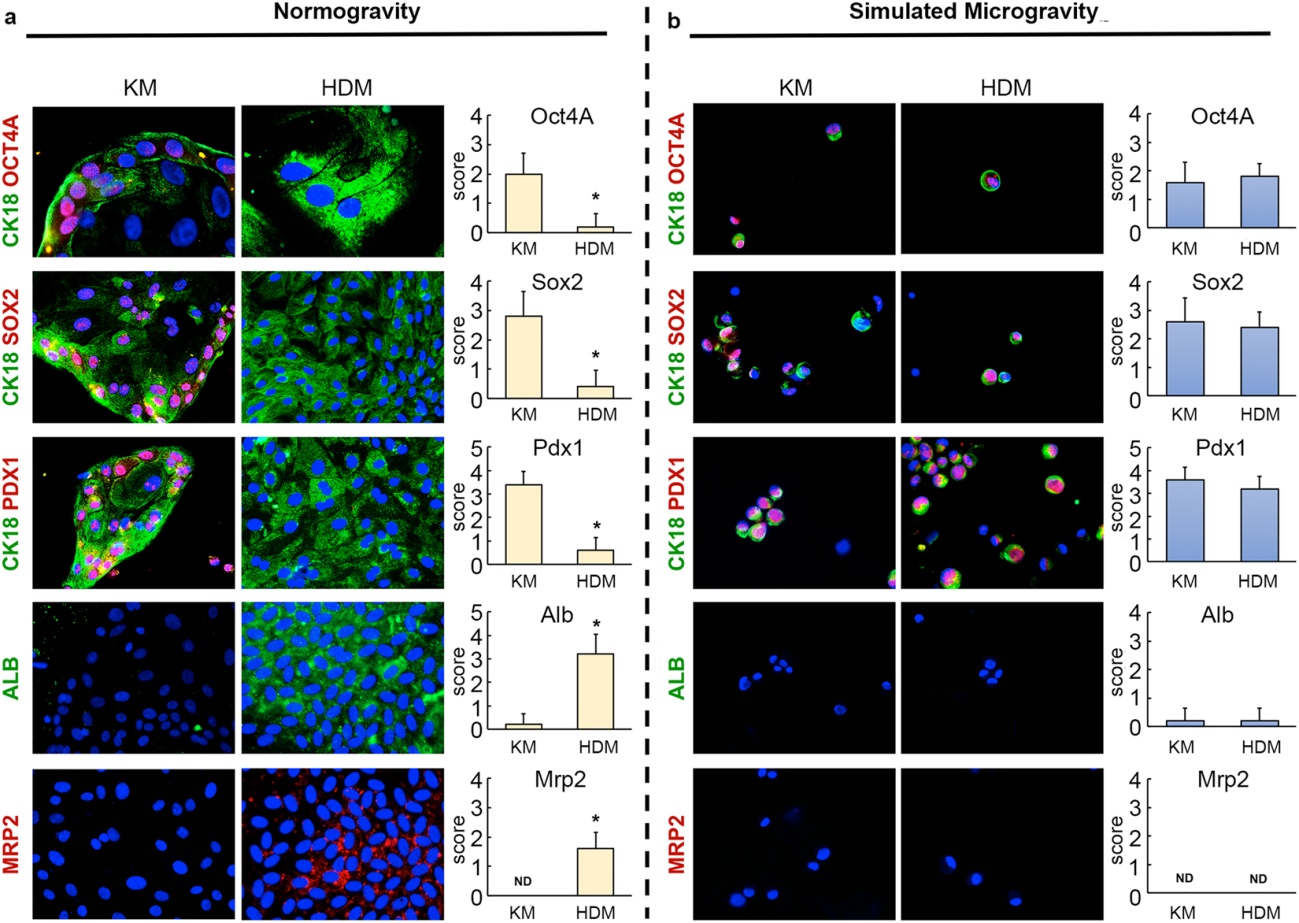
Comparing the expression of stem cell and mature hepatocyte markers in hBTSCs, cultured in normogravity (NG) and simulated microgravity (SMG) in different media. Expression of stem cells and mature hepatocyte markers was analyzed by immunofluorescence. hBTSCs cultured in KM in NG expressed OCT4, SOX2, PDX1 (red) and CK18 (green), when cells cultured in HDM expressed ALB (green) and MRP2 (red) (Magnification 20X) (**a**). hBTSCs cultured both in KM and in HDM expressed similar levels of OCT4, SOX2, PDX1 (red) in SMG, but no expression of ALB (green) and MRP2 (red) (Magnification 20X) (**b**).

In order to achieve semi-quantitative data on the protein expression of the modulated genes, cells were removed from the vessels and smeared on slides to perform immunofluorescence analysis. Because of this technical aspect, cell cultures did not retain their 3D organization, which did not reflect the appearance of the colony under microgravity. However, the immunofluorescence analysis confirmed in NG condition the reduced expression of stem cells markers (Oct4A, Sox2, Pdx1) (p < 0.05) and the increased expression of mature markers (Alb, Mrp2) (p < 0.05) in hBTSCs maintained in HDM than cells under self-replication condition (KM) (Fig. 4a). No differences were observed in the expression of the same markers in hBTSCs cultivated in SMG in the two different culture media (Fig. 4b). Moreover, when hBTSCs were cultured in HDM and in SMG, they showed a scarse cytoplasm and a high nucleus-to-cytoplasm ratio (features of stem cells) while in NG condition cells had a large cytoplasm.

**Figure 5:**
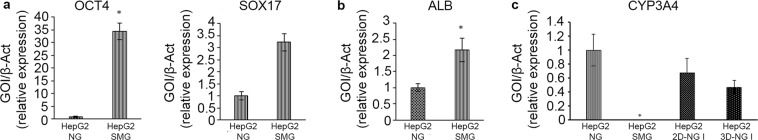
Transcriptional analysis of HepG2 cells in simulated microgravity (SMG). Levels of gene expression of two stem cell markers (OCT4 and SOX17) (**a**) and a mature hepatocyte marker (ALB) in HepG2 cells cultured in NG compared to cells cultured on Cytodex 3 microcarriers in SMG (**b**). The gene expression of CYP3A4 in HepG2 cells cultured on Cytodex 3 microcarriers in SMG significantly dropped down (*p < 0.05; n = 4) compared to NG controls. Levels of CYP3A4 transcription were recovered when cells were put back in NG conditions, both cultured as a monolayer (2D) and on Cytodex 3 microcarriers (3D) (**c**).

**Figure 6:**
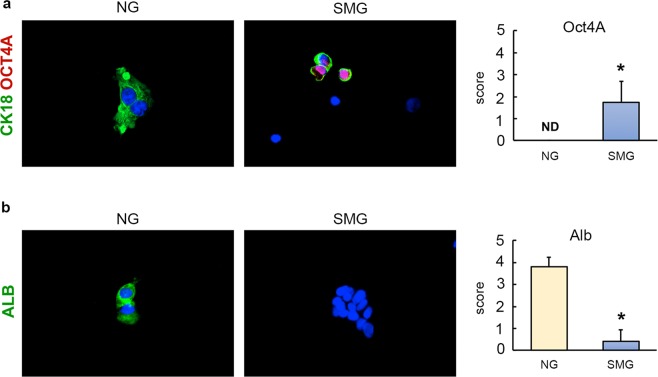
Protein expression of OCT4A and ALB in HepG2 cultured in normogravity (NG) and simulated microgravity (SMG). Expression of stem cells and mature hepatocyte markers was analyzed by immunofluorescence. HepG2 cultured in the specific medium expressed nuclear OCT4 (red) (**a**) while no expression of ALB (green) (**b**) was revealed in SMG condition (Magnification 20X).

HepG2 were cultured in their canonical HDM medium both in NG and SMG conditions. For HepG2 cells low or null expression of stem cell genes, such as OCT4 and SOX17, were detected as expected in NG. Interestingly, we observed a significant increased expression of these stem cell genes in SMG (*p* < 0.05; Fig. 5a). Furthermore, SMG, compared to NG, stimulated ALB gene expression in HepG2 cells (p < 0.05; Fig. 5b), and the reduction of the expression of CYP3A4 gene, a marker of late-stage (i.e. Zone 3) hepatocytes (p < 0.05; Fig. 5b). Notably, CYP3A4 expression was reacquired at NG condition levels when cells were transferred from SMG to NG (p < 0.05 vs SMG; Fig. 5c). Immunofluorescence analysis demonstrated that SMG condition enhanced the expression of pluripotent stem cell marker such as Oct4a (p < 0.05 vs NG; Fig. 6a) and revealed low level of albumin (p < 0.05 vs NG; Fig. 6b). Therefore, SMG reduced the acquisition of a late-stage hepatocyte phenotype in HepG2 cells.

### Analysis of exo-metabolome by nuclear magnetic resonance (NMR)

**Figure 7:**
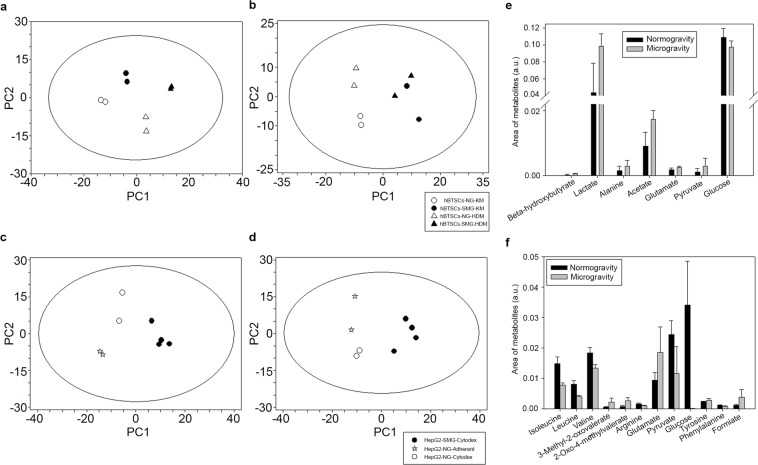
NMR and loading analysis. Overview of the principal components analysis (PCA) model built on the p-JRES NMR dataset of media samples of hBTSCs cultured in Kubota’s Medium (KM) or hormonally defined medium (HDM) in normal gravity (NG) or simulated microgravity (SMG) at day 3 (**a**) and 13 (**b**). Analyses were performed for media samples of HepG2 cells cultured in NG or SMG condition at day 3 (**c**) and 13 (**d**). The score plots (**a**–**d**) show the differences among samples, while the loading plot highlights which metabolites are responsible in separating the NG and SMG samples in hBTSCs and HepG2 cells respectively (**e**,**f**). All metabolite data were statistically significant (p < 0.05).

^1^H-NMR spectra of media from hBTSCs (Fig. 7a,b) and HepG2 cell cultures (Fig. 7c,d**)** in KM or HDM and, in NG or SMG condition were analyzed using the Principal Component Analysis (PCA).

Starting from 3 days in culture, significant metabolic differences were observed in hBTSCs in KM than in HDM media, Specifically, on the principal component 1 or PC1 (*p* = 0.005) that is the linear combination of the starting variables with the maximum variance; while on the principal component 2 or PC2, the linear combination of the variables with the immediately inferior variance and orthogonal to PC1, it was possible to observe only significant differences between cells grown in NG or SMG condition (*p* = 0.02) (Fig. 7a).

With the increase of experimental time, the metabolic effects due to SMG condition became more evident: we observed differences between samples from SMG cells and their control cells in NG condition at day 8 on PC2 (*p* = 0.01) (not showed) and at day 13 on PC1 (*p* < 0.00001) (Fig. 7b). Moreover, at day 13, we observed metabolic differences between medium samples from cells cultured in KM or HDM only in NG condition. Then, to identify the metabolites that best contribute to the discrimination between samples from SMG cells and their control cells in NG condition at day 13 on PC1, we analyzed the loadings values from PCA with a threshold of 0.95 (Fig. 7e). PC1 included the following variables with the highest correlation levels: beta-hydroxybutyrate, lactate, alanine, acetate, glutamate, pyruvate and glucose. Therefore, considering the net balance of these metabolites (i.e. the difference between all the bins of the spectra acquired at day 13 and the corresponding averaged bins of the spectra at 0 hours), PC1 indicated that the SMG induced a lower consumption of glutamate and pyruvate as well as a higher consumption of glucose. Moreover, the cells in SMG condition increased the production of beta-hydroxybutyrate, lactate, alanine and acetate. Thus, the SMG increased the glycolytic phenotype of hBTSCs.

The analysis of HepG2 culture medium evidenced an analogue difference between the metabolism dependent on NG/SMG condition: we observed significant metabolic differences between cells cultured in NG (both adherent and Cytodex 3 cultures) or SMG condition on PC1 at each experimental time (*p* < 0.00001) (Fig. 7c,d). The cells cultured in SMG condition showed a lower consumption of glutamate and tyrosine as well as a higher consumption of branched amino acids, arginine, pyruvate, glucose and phenylalanine. These cells also produced more 3-Methyl-2-oxovalerate, 2-oxo-4-methyl-valerate and formiate with respect to controls (Fig. 7f). As for hBTSCs, the SMG induced a glycolytic phenotype in HepG2 cells.

## Discussion

SMG favors the development of tridimensional cultures of hBTSCs and HepG2. Indeed, hBTSCs and HepG2 were able to grow in SMG adhering to the surface of Cytodex 3 where large and wide cell clusters were formed into a higher extent than in traditional bi-dimensional plated cultures.

SMG stimulated cell-cell and cell-matrix interactions^15,16^ and that facilitated the formation of tridimensional clusters, in association with favoured cell proliferation. Interestingly, different cell lineages growing in microgravity undergo a spontaneous transition leading to the emergence of two distinct phenotypes, while, the return in a normal gravitational field, the two phenotypes collapse, recovering their original configuration^2^. It has been hypothesized that, once the gravitational constraint is removed, the system freely explores its phenotypic space^2^. The optimized 3D growth observed in the simulated microgravity in hBTSCs and HepG2 is in agreement with the theory of the lack of the gravitational constraint. The improvement of SMG-associated cultures could allow the maintaining of vital tissues and organ sections to be potentially destined to clinical therapy^17^.

Furthermore, our study demonstrated an important impact of SMG on both gene and protein expression and metabolome in hBTSCs and HepG2 cells. Gene expression and protein analysis suggested a role of SMG in maintaining stemness and counteracting cell differentiation, while, exo-metabolomic analysis demonstrated a role of SMG in stimulating a glycolytic metabolism. Indeed, when hBTSCs were cultured in HDM, that should induce differentiation toward mature hepatocytes, stemness was maintained by SMG as demonstrated by cell morphology and both gene and protein expression analyses.

Interestingly, SMG-induced maintenance of stemness was associated with a predominant glycolytic metabolism in hBTSCs. These effects of SMG were not cell-specific since were also observed in HepG2 cells, where SMG reduced the expression of CYP3A4, a marker of late-stage (i.e. Zone 3) hepatocytes. Interestingly, at both gene and protein level expression, in SMG it has been revealed the expression of a pluripotency marker, such as Oct4a, in HepG2 cells. As far as albumin expression, it is noteworthy that the gene and protein expression analysis showed an opposite trend. Indeed, while albumin is expressed in NG, as expected, and reduced in SMG, the albumin gene resulted highly transcribed in SMG vs NG. This apparent discordance may be explained by the significance of the albumin itself, which is an early-stage hepatocyte gene, and thus expressed solely as mRNA by hepatic/stem progenitor cells or hepatoblasts, while stably expressed as protein in mature hepatocytes^18–21^. Thus, the phenotype of HepG2 in SMG is consistent with the one of hepatic/stem progenitor cells^18–21^. Finally, also in HepG2 cells transcriptomic modulation exerted by SMG is associated with the induction of a glycolytic metabolism. This was demonstrated by the analysis of the metabolic loading, by NMR spectra, performed in either hBTSCs or HepG2 cells under SMG, irrespectively from the culture media.

Different studies showed that pluripotency of human embryonic stem cells is associated with polyunsaturated metabolome^22^. Notably, glycolytic cell metabolism and the resulting metabolome have been associated with stemness and pluripotency in human pluripotent stem cells^23^ while the differentiation processes, associated with maturation of the mitochondrial network, were characterized by a metabolic shift toward oxidative phosphorylation1^24,25^. It is of interest that a glycolytic metabolism in aerobic condition is typical of tumour cells, and this is known as “Warburg Effect”^18^. In keeping, CCA primary cell lines, the neoplastic counterpart of biliary epithelium characterized by high representation of cancer stem cells showed a metabolic profile similar to hBTSCs maintained in SMG condition (data not shown).

A point of high relevance for 3D structures has been noted and concerns the experience of oxygen and nutrients gradients in 3D strictures, with decreasing values from the outer surface to the inner core of the cell cluster. Notably, the system adopted here was the object of a deep study of the diffusion of oxygen and nutrients into the rotating vessel by Ayyaswamy P.S. & Mukundakrishnan K^26^. Authors demonstrated that the environment created within the vessel during the experiment is characterized by a stable diffusion of O2 and nutrients^26^. In our paper, to confirm the lack of a metabolic sufferance of 3D aggregates keep in cultures under microgravity we have analyzed the supernatant of the vessel and compared data on glucose consumption with the same cells keep in normogravity, which showed that the level of glucose in the supernatant of hBTSCs growth under microgravity as 3D organoids or in that of hBTSCs growth in normogravity in standard cell culture system do not differ each other. Although these data would exclude that the observed changes in gene expression and metabolism may depend on oxygen and nutrients availability, rather than by microgravity alone, further studies should be performed, e.g. using cells cultured in dynamic conditions using a microfluidic device.

Previously, it has been demonstrated that the SMG induces a decreased in the activity and/or gene expression of enzymes involved in oxidative energy production in muscles, while, in contrast, glycolytic metabolism seemed to be slightly increased in human adult subjected to experimental conditions, mimicking long term MG by resting in 6° head-down tilt position for 84 days^27^.

The transduction of the gravity signal on which its biologic effects rely on is currently under investigation^28,29^. Extracellular mechanic stimuli, capable to modulate cell metabolism and mitochondria, have been described primarily in plant cells^28,29^. In this contest the sensorial apparatus has been identified in membrane receptors, cytoskeletal, and intracellular messengers linked to the modulation of metabolic enzymes^28,29^. Aryl hydrocarbon receptor (AHR) a member of the basic-helix/loop/helix per-Arnt-sim (bHLH/PAS) family of “sensors” of foreign and endogenous signals, has been implicated in gravity sensation. Interestingly, its stimulation up-regulated cytochrome P450 family 1 (CYP1) and another enzyme activities^28^. The absence or reduction of gravity may downregulate the AHR mediated metabolic enzyme activation. Our paper warrants future investigations to unveil the mechanisms which link the SMG to metabolome modulation in human cells, especially in stem cells, which are naturally highly sensitive to metabolomic changes.

In conclusions, our results could be implemented by developing SMG-based platforms for scale production of large number of hBTSCs which in turn can found applications for *in vitro* texting and modelling (e.g. liver toxicity tests), regenerative medicine of liver, or production of large amount multipotent stem cell population. Finally, the effects of microgravity on stem cell biology in subjects working in this condition should be contemplated in space medicine programs due the demonstrated impact of SMG on liver health. To this latter regard, results of this paper constitute the basis to develop further studies and technologies where hBTSCs maintained in SMG will be investigated to identify mechanisms and putative therapeutic targets to modulate stem cell differentiation.

## Material and Methods

### Human tissues collection

All the human extrahepatic biliary trees, including common hepatic duct, bile duct, cystic duct, gallbladder, and hepato-pancreatic ampulla which have been used in this work were obtained from organ donors from the “Paride Stefanini” Department of General Surgery and Organ Transplantation, Sapienza University of Rome, Rome, Italy. Informed consent to use tissues for research purposes was obtained from our transplant program. All samples derived from adults between the ages of 19 and 73 years. No organs or tissues were procured from prisoners. Protocols received the approval of our Institutional Review Board, and the processes were compliant with current Good Manufacturing Practice (cGMP). The research protocol was reviewed and approved by the Ethic Committee of Umberto I University Hospital, Rome. Each set of experiments was performed on cells isolated from four different patients, in triplicate for each patient.

### Tissues processing

Cells from tissues samples were isolated as described previously^13,14,19,26^. Briefly, after a macroscopic and microscopic exam in sterile condition, tissues were washed in DPBS (Gibco, catalog #14190-094) and were digested in RPMI-based digestion buffer enriched with 0.1% human serum albumin, 1 nM selenium, antibiotics, type I collagenase (300 collagen unit/ml), 0,3 mg/ml deoxyribonuclease, at 37 °C, with frequent agitation for 30–45 minutes to be properly processed later. Then, cells were progressively filtered by sterile 100 µm and 30 µm strainers to remove greater residues.

### Epithelial cell adhesion molecule (EpCAM) sorting procedures

Cells were immunosorted for EpCAM marker by specific magnetic beads. As specified by the manufacturer (Miltenyi Biotec Inc., Germany), EpCAM+ cells were magnetically labeled with EpCAM antibody-coated microbeads (Miltenyi Biotec Inc., catalog #130-061-101) and loaded onto a MACS LS Column (Miltenyi Biotec Inc., catalog #130-042-401). Separation process was performed in presence of a magnetic field generated by a MACS Separator. EpCAM+ cells were counted and suspended in the corresponding medium at a concentration of 300,000 cells per ml, and then used as the final cell suspension to be cultured in the different gravity condition and growth supports.

### Non-adherent cultures

EpCAM+ hBTSCs have been isolated and split out to be cultured both in SMG and NG condition. Both stem and hepatocyte differentiation culture media have been enriched with B27 (ThermoFisher Scientific, catalog #12587010) and bFGF (Gibco, catalog #13256029) molecules to maintain cells in a non-adhesion culture state.

### Culture with Cytodex 3

EpCAM+ hBTSCs have been split out to be cultured in both NG and SMG. Culture parameters were optimized for hBTSCs cultures. Cytodex 3 microcarriers have been first rehydrated for at least 3hrs and they were sterilized by autoclave for 15′ at 121 °C according to the producer’s directions (Sigma-Aldrich, Milan, Italy, catalog #C3275) and cells have been incubated for at least 30 minutes in presence of Cytodex 3 at 37 °C and 5% CO_2_ to favour their interaction and adhesion with their dextran inert spherical substrate.

### Media and solutions

Cells in these experiments were cultured in several media. All media were filtered by sterile 0.22 µm strainer and kept in the dark at 4 °C. In particular, RPMI-1640 and MEM EBS the basal media of respectively biliary stem progenitor cells and HepG2, and foetal bovine serum (FBS) were obtained from Gibco, Life Technologies, Milan, Italy. All reagents were obtained from Sigma-Aldrich, Milan Italy, unless otherwise specified.

### Kubota’s Medium

Human endodermal stem cells were cultured in the Kubota’s Medium, a serum-free basal medium that demonstrated to be suitable for human HpSCs^12,18^, hBTSCs^13,17,20,24,26^, human pancreatic stem/progenitor cells^20^, and rodent HpSCs^25^. This special medium is prepared with RPMI 1640 added with no copper, low calcium (0.3 mM) and other compounds, as described more in detail by the protocol of Kubota and Reid^20^. No mature epithelial cells of liver, biliary tree, and pancreas can survive in this medium. Eventual co-isolated mesenchymal cells disappear after 6–10 days of culture.

### Hormonally defined medium (HDM)

A tailored modified Kubota’s medium was used to induce cells to differentiate in mature hepatocytes as demonstrated elsewhere^17,21,24^. Serum-free Kubota’s Medium was supplemented with calcium (final concentration 0.6 mM), copper (10–12 M) and 20 ng/mL basic fibroblast growth factor (bFGF). Furthermore, 7 μg/L glucagon, 2 g/L galactose, 1 nM triiodothyroxine 3 (T_3_), 10 ng/mL Oncostatin M (OSM), 10 ng/mL epidermal growth factor (EGF), 20 ng/mL hepatocyte growth factor (HGF), and 1 µM dexamethasone were added to modified Kubota’s medium to prepare the final Hormonally defined medium (HDM).

### Loading of vessels and cell cultures in SMG condition

Cells have been dispersed in a 10 ml of media (Kubota’s medium for hBTSCs, HDM for hepatocyte differentiation of hBTSCs, HepG2 medium) and then inoculated through the opportune borehole inside the 10 ml disposable culture vessels (Cellon S.A., catalog #D-410) of the Rotary Cell Culture System (RCCS-4D) from Synthecon. Vessel rotation was optimized for hBTSCs culture. It was set into a range of 17–24 rpm, on the basis of cluster formation and their dimensions, and maintained for the whole time of the experiment.

At every medium change, samples of culture media have been accurately withdrawn to be maintained for the analysis of the exo-metabolome.

### Cell cultures in NG condition

Sorted EpCAM+ cells (approximately 3 × 10^5^ per well), isolated from biliary tissue specimens, and HepG2 cells were seeded onto 3 cm diameter plastic culture dishes. For the analysis of mature markers like CYP3A4 after SMG culture, hBTSCs were put back in NG condition for 15 days in presence of HDM in two different ways: as monolayer (2D-NG) and attached to the Cytodex 3 microcarriers (3D-NG) in plastic culture dishes as above. Similarly, HepG2 cells were cultured in NG for 10 days in 2D-NG and 3D-NG in the specific medium after their period in SMG.

### Analysis of viability by Trypan Blue assay

Living cells have been esteemed by Trypan Blue assays (Sigma-Aldrich, catalog #T8154-100ML). Trypan blue is a selective dye able to discriminate living cells (white) from non-living ones (blue). This dye was used 1:1 v/v with cell solution. Counts were performed by a FAST-READ 102 (Biosigma) and viability has been calculated as the percentage of living cells respect the total number of cells.

### RNA extraction and Reverse-Transcription polymerase chain reaction

Total nucleic acids were extracted from cell cultures according to the procedures of Chomczynski and Sacchi^30^. Cells have been detached from their supports and then collected in a TRIzol solution (Life Technologies; Cat# 15596–026) for the extraction. Total mRNA was quantified by Nanodrop (Thermo Fisher Scientific Inc., USA). Gene expression analyses were performed by reverse-transcription and cDNA amplification in a closed tube (OneStep RT-PCR by Qiagen, Hamburg, Germany) on total RNA samples extracted from cells. The expression of each gene of interest was calculated by the ratio of the concentrations of the genes of interest and the reference gene B-Act (reported by instrument in nmol/L) (Supplementary Table 1).

### Immunofluorescence analysis

Vessel was stopped for 60 minutes in order to harvest the cells inside the vessel in SMG condition.

In order to achieve semi-quantitative data, cells were removed from the vessel, were counted and, finally, were smeared on a slide to perform immunofluorescence analysis. Cells from NG and SMG conditions were fixed in pure acetone for 10 minutes at room temperature to be analyzed by immunofluorescence. Because of technical aspects, cell cultures did not retain their 3D organization, which did not reflect the appearance of the colony under microgravity. Primary antibodies against OCT4A (#2050; Cell Signaling), SOX2 (AB97959; Abcam), PDX1 (SC-25403; Santa Cruz), ALB (F0117; DAKO) and MRP2 (ab3373; Abcam) were used. Then, cells were washed and incubated for 1 hour with labeled isotype specific secondary antibodies (anti-mouse AlexaFluor-488, anti-rabbit Alexafluor-546, Invitrogen, Life Technologies Ltd, Paisley, UK) and 4,6′-diamidino-2-phenylindole (DAPI) was used to counterstain cell nuclei. Protein expression were examined by Leica Microsystems DM 4500 B Light and Fluorescence Microscopy (Weltzlar, Germany) equipped with a JenoptikProg Res C10 Plus Videocam (Jena, Germany) (Supplementary Table 2).

### Sample preparation for NMR spectroscopy

Each medium sample (2 ml) was lyophilized then dissolved in 700 μl of 1 mM TSP [sodium salt of 3-(trimethylsilyl) propionic-2,2,3,3-d4 acid], 10 mM sodium azide D_2_O phosphate buffer solution (pH = 7.4) and finally homogenized by vortex mixing for 1 min. After centrifugation (10 min, 10,000 RCF at 22 °C), 600 μl of each resulting supernatant was transferred to a 5-mm NMR tube and used for the NMR analysis.

### ^1^H-NMR Spectroscopy

2D 1H J-resolved (JRES) NMR spectra were acquired on a 500 MHz Varian/Agilent spectrometer (Agilent, Santa Clara, CA) using a double spin echo sequence with 4 transients per increment for a total of 32 increments. These were collected into 16 k data points using spectral widths of 6 kHz in F2 and 40 Hz in F1. There was a 2.0 s relaxation delay. Each FID was Fourier transformed after a multiplication with sine-bell/exponential function in the F2 dimension and a sine-bell function in the F1 dimension. JRES spectra were tilted by 45°, symmetrised about F1, referenced to TSP at dH = 0.0 ppm and the proton-decoupled skyline projections (p-JRES) exported using Agilent VNMRJ 3.2 software. Metabolites responsible for the separation between samples from cells cultured in SMG or NG condition were identified using an in-house NMR database and Chenomx NMR suite v. 7.7 (Chenomx Inc., Alberta, Canada).

### NMR spectra analysis

The 1D skyline projections exported were aligned and then reduced into spectral bins with widths ranging from 0.01 to 0.02 ppm by using the ACD intelligent bucketing method [1D NMR Manager software (ACD/Labs, Toronto, Canada)]. To compare the spectra, the integrals derived from the binning procedure were normalized to the total integral region, following exclusion of bins representing the residual water peak (d 4.33–5.17 ppm) and the TSP peak (d 0.5–0.5 ppm). The resulting data was used as input for Principal Component Analysis (PCA)^31^ performed using SIMCA-P+ version 12 (Umetrics, Umea, Sweden). The PCA analysis is described in the method section of Cioce *et al*. paper^32,33^.

### Ethical approval and informed consent

Informed consent to use tissues for research purposes was obtained from our transplant program. All samples derived from adults between the ages of 19 and 73 years. All experimental protocols were approved by Sapienza University/Policlinico Umberto I Institutional Review Board, and processing was compliant with current Good Manufacturing Practice (cGMP).

## Supplementary information

Supplementary Info

Supplementary video 1

Supplementary video 2

## Data Availability

All materials, data and associated protocols are promptly available to readers without undue qualifications in material transfer agreements.
