# Supplementary material for: Simulated microgravity promotes the formation of tridimensional cultures and stimulates pluripotency and a glycolytic metabolism in human hepatic and biliary tree stem/progenitor cells
Source: Sci Rep. 2019 Apr 3;9:5559. doi: 10.1038/s41598-019-41908-5 (PMC6447605; doi:10.1038/s41598-019-41908-5)
Supplement: Supplementary file 1 — Supplementary Info [file 41598_2019_41908_MOESM1_ESM.pdf]

Simulated microgravity promotes the formation of tridimensional cultures and stimulates pluripotency and a glycolytic metabolism in human hepatic and biliary tree stem/progenitor cells.

Daniele Costantini, Diletta Overi, Luca Casadei, Vincenzo Cardinale, Lorenzo Nevi, Guido Carpino, Sabina Di Matteo, Samira Safarikia, Mariacristina Valerio, Fabio Melandro, Mariano Bizzarri, Cesare Manetti, Pasquale Bartolomeo Berloco, Eugenio Gaudio, Domenico Alvaro

SUPPLEMENTARY TABLES

Supplementary Table 1. Table of human qPCR primer pairs for gene expression.

Gene	Gene Bank	Forward Primer	Reverse Primer
OCT4	NM_002701	TCGAGAACCGAGTGAGAGG	GAACCACACTCGGACCACA
SOX17	NM _022454	AAGATGCTGGGCAAGTCGTGG	CTTGTAGTTGGGGTGGTCCTG
PDX1	NM_000209	CATTGGAAGGCTCCCTAACA	TTCCACTGGCATCAATTTCA
SOX2	NM_003106	GGGAAATCCCAGGGGTGCAAAAGAGG	TTGCGTGAGTGTGGATGGGATTGGTG
ALB	NM_000477.5	AGAGGTCTCAAGAAACCTAGGAAA	GGTTCAGGACCACGGATAGA
CYP3A4	NM_017460	AAGTCGCCTCGAAGATACACA	AAGGAGAGAACACTGCTCGTG
β-Actin	NM_007393.5	GGATGCAGAAGGAGATTACTGC	CCACCGATCCACACAGAGTA

The ratio of concentration of gene of interest and the reference gene human β-Actin was assumed to be the gene of interest relative expression.

Supplementary Table 2. List of used antibodies and their application(s)

Name	Host / isotype	Source	Catalog#	Dilution	Application
Albumin	Rabbit IgG	Abcam	Ab2406	1:200	IF
K19 (cytokeratin 19)	Mouse IgG1	DAKO	M0888	1:100	IF
MRP2	Mouse IgG2a	Abcam	ab3373	1:200	IF
OCT4A	Rabbit IgG	Cell Signaling	#2050	1:50	IF
PDX1	Rabbit IgG	Santa Cruz	SC-25403	1:50	IF
SOX2	Rabbit IgG	Abcam	AB97959	1:200	IF
SOX17	Goat IgG	R&D	AF1924	1:50	IF

SUPPLEMENTARY VIDEO LEGEND

Supplementary video 1. This video showed the formation of a real organoid having a considerable size. The organoids were continuously in free fall and were affected by microgravity conditions.

Supplementary video 2. In the video it possible to observe smaller structures which are clearly suspended and for which the rotation speed had been correctly modulated to keep them in suspension.

SUPPLEMENTARY FIGURES

Supplementary Figure 1

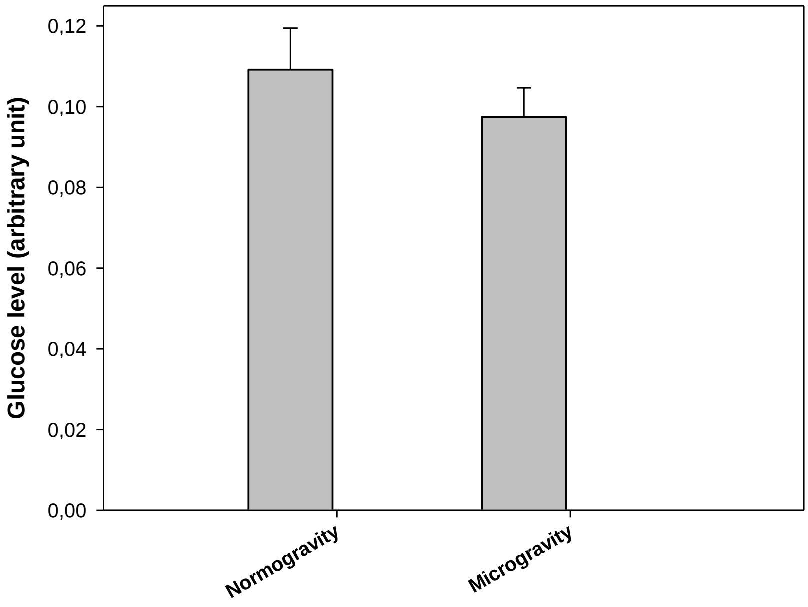

Supplementary Figure 1. Glucose levels into the grow medium in normogravity and microgravity conditions. Glucose levels of hBTSCs in microgravity analysed did not show significative difference (N=3; p> 0.05) when compared to glucose levels of hBTSCs cultured in normogravity.

Supplementary Figure 2

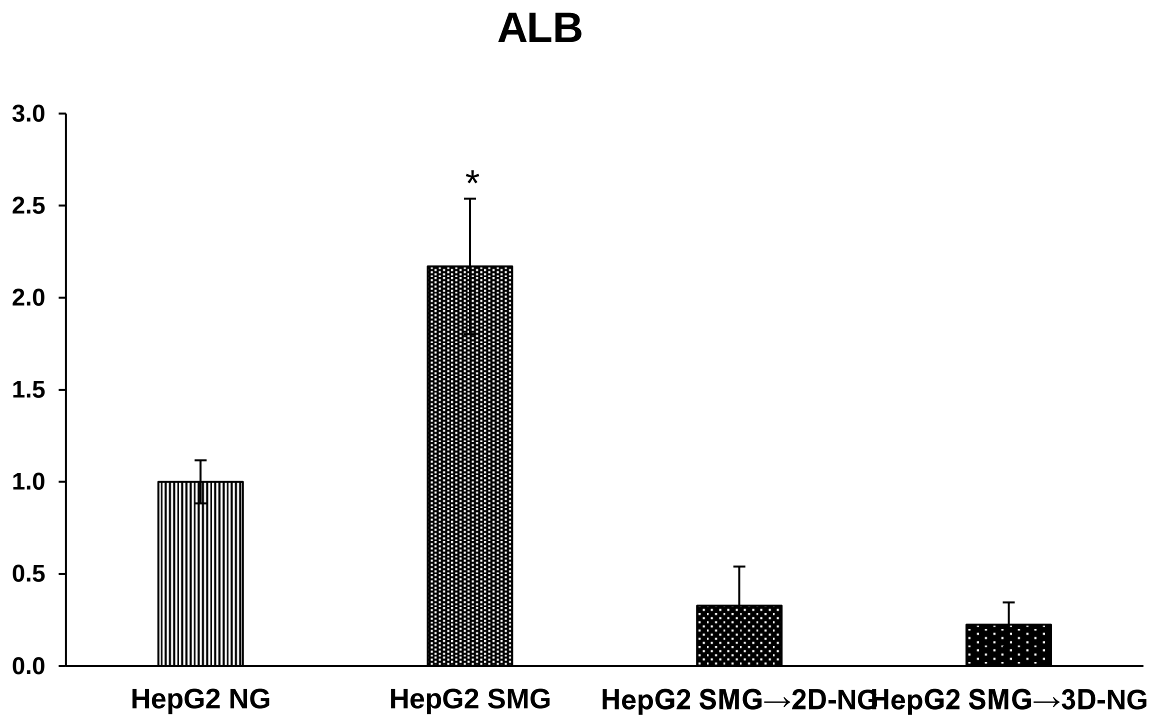

Supplementary Figure 2. Albumin gene expression into the in normogravity and simulated microgravity conditions. Graphs illustrating the gene expression of albumin (N=3, $p < 0.05$) in HepG2 cells have been reported in different conditions, in normogravity (NG), simulated microgravity (SMG), and in cells maintained in NG (2D or 3D growth) for 15 days after being cultured in SMG (SGM→2D NG and SGM→3D NG).
